# Ceramide Is Involved in Temozolomide Resistance in Human Glioblastoma U87MG Overexpressing EGFR

**DOI:** 10.3390/ijms242015394

**Published:** 2023-10-20

**Authors:** Rosaria Bassi, Michele Dei Cas, Cristina Tringali, Federica Compostella, Rita Paroni, Paola Giussani

**Affiliations:** 1 Department of Medical Biotechnology and Translational Medicine, Università degli Studi di Milano, LITA Segrate, Via Fratelli Cervi, 93, 20090 Segrate, Italy; 2 Department of Scienze della Salute, Università degli Studi di Milano, Via di Rudini, 8, 20142 Milan, Italy

**Keywords:** ceramide, sphingolipids, epidermal growth factor receptor, cell survival

## Abstract

Glioblastoma multiforme (GBM) is the most frequent and deadly brain tumor. Many sphingolipids are crucial players in the regulation of glioma cell growth as well as in the response to different chemotherapeutic drugs. In particular, ceramide (Cer) is a tumor suppressor lipid, able to induce antiproliferative and apoptotic responses in different types of tumors including GBM, most of which overexpress the epidermal growth factor receptor variant III (EGFRvIII). In this paper, we investigated whether Cer metabolism is altered in the U87MG human glioma cell line overexpressing EGFRvIII (EGFR+ cells) to elucidate their possible interplay in the mechanisms regulating GBM survival properties and the response to the alkylating agent temozolomide (TMZ). Notably, we demonstrated that a low dose of TMZ significantly increases Cer levels in U87MG cells but slightly in EGFR+ cells (sensitive and resistant to TMZ, respectively). Moreover, the inhibition of the synthesis of complex sphingolipids made EGFR+ cells sensitive to TMZ, thus involving Cer accumulation/removal in TMZ resistance of GBM cells. This suggests that the enhanced resistance of EGFR+ cells to TMZ is dependent on Cer metabolism. Altogether, our results indicate that EGFRvIII expression confers a TMZ-resistance phenotype to U87MG glioma cells by counteracting Cer increase.

## 1. Introduction

Glioblastoma multiforme (GBM) is the most common and malignant primary brain tumor in humans [1,2,3]. According to the World Health Organization (WHO), GBMs are grade IV tumors characterized by extended vascularization and high invasiveness, as well as extensive infiltrative growth [1]. These features contribute to the very poor prognosis of GBM, with a median survival of 12–18 months from the diagnosis despite aggressive treatments mainly consisting of a combination of surgery followed by radiotherapy and chemotherapy with the methylating agent temozolomide (TMZ) [1].

Several studies in the past three decades have provided strong evidence that different sphingolipids (SLs) are the key signaling mediators involved in the control of cell proliferation and the fate in several physio-pathological conditions [4,5,6,7,8]. In particular, it has been widely demonstrated that ceramide (Cer), the central metabolite of SL pathways, is a tumor suppressor lipid, which is able to induce antiproliferative and apoptotic responses, thus playing a crucial role in cancer progression and in the mechanisms of action of many chemotherapeutic drugs [8,9,10,11,12,13,14]. Indeed, altered Cer metabolism is a common feature in several types of tumors including GBM. Interestingly, Cer levels are inversely associated with malignant progression and poor prognosis of GBM [11]. Moreover, different studies have suggested that evading Cer-mediated apoptosis is also critical to drug resistance, and raising Cer levels may offer a potential approach to treat cancer patients by overcoming chemotherapeutic resistance [13,15,16]. For example, increasing Cer levels by inhibiting glucosylceramide (GlcCer) synthase has been shown to induce death in multidrug resistance (MDR) cells as well as to enhance the proapoptotic activity of different anticancer agents in human cancer cells including glioma cells [4,17,18]. In addition, blocking the conversion of Cer to sphingomyelin (SM) or its degradation to sphingosine by inhibiting SM synthase or ceramidase, respectively, have also been related to an increase in the antitumoral effect of several drugs through Cer-activated cell death pathways [19,20].

On the other hand, one of the factors linked to poor survival in GBM patients is the expression of the epidermal growth factor receptor variant III (EGFRvIII), the most common EGFR gene mutation in GBM. EGFRvIII encodes for a truncated form of the receptor lacking the extracellular binding domain, resulting in ligand-independent constitutive tyrosine kinase activity. Consequently, aberrant alterations in down-stream signal transduction pathways, such as phosphatidylinositol-3 kinase (PI3K)/Akt and Ras/MEK/ERK, can lead to uncontrolled proliferation and invasion of glioma cells [21]. EGFR has been demonstrated to be a diagnostic and, although controversial, prognostic marker in human GBMs. It has been demonstrated that EGFR plays a role in gliomagenesis but also in tumor cell motility and invasiveness [22]. The PI3K/AKT signaling pathway, which is involved in the regulation of GBM cell survival, is known to be hyperactivated by EGFR [23]. Increasing evidence has demonstrated that Cer acts as a negative regulator of the PI3K/AKT pathway [24,25,26,27], which in turn represses apoptosis and autophagy [15].

In this study, we investigate Cer metabolism related to EGFRvIII expression to elucidate their possible interplay in the mechanisms regulating the survival properties and the drug response in GBM. Our results indicate that EGFRvIII expression contrasts the toxic action of TMZ in GBM cells, counteracting Cer increase.

## 2. Results

### 2.1. EGFRvIII Expression Correlates with Higher Survival Rate of GBM Cells

**Figure 1:**
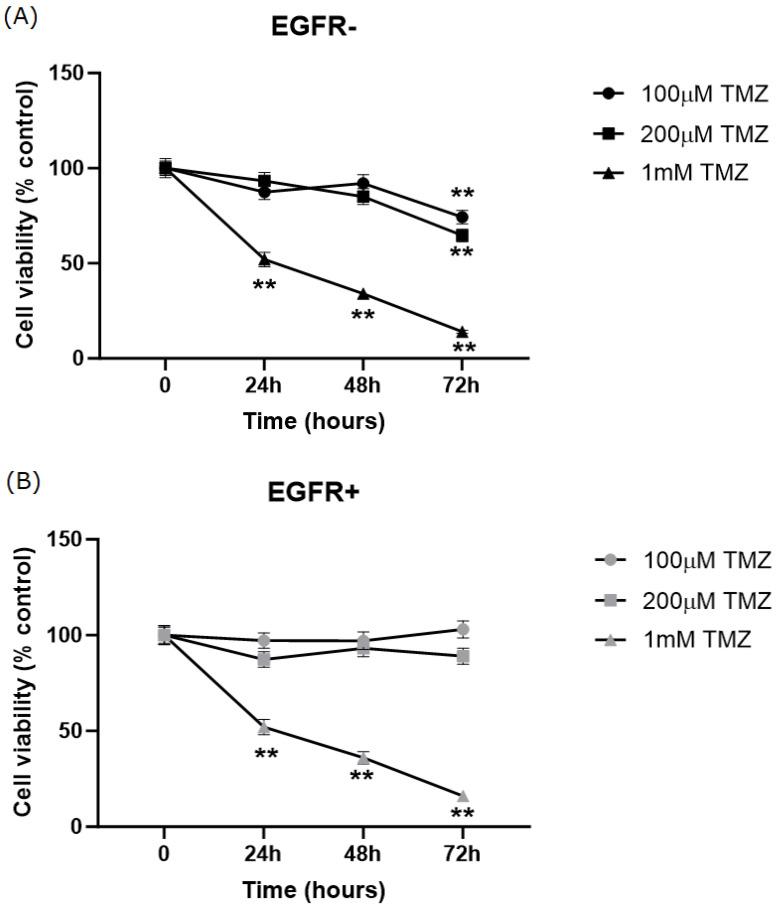
Effect of TMZ on survival properties of EGFR− and EGFR+ cells. EGFR− (**A**) and EGFR+ (**B**) cells were seeded at 20,000 and 30,000 cells/cm^2^, respectively, and exposed to different doses of TMZ (0.1–1 mM) for different times: 24 h, 48 h, and 72 h. Cell viability was assessed by MTT assay at the end of treatment. Results are expressed as percentage of cell survival with respect to vehicle-treated cells. Data are mean ± SD of three independent experiments. ** *p* < 0.01 versus EGFR− cells or EGFR+ cells (one-way ANOVA followed by Tukey’s post hoc test).

In addition to supporting invasiveness, EGFR signaling is known to promote cell proliferation and survival [22,28]. Therefore, we evaluated the influence of EGFR expression on GBM cell survival by comparing the survival rate of EGFR+ (EGFRvIII overexpressing U87MG cells) and EGFR− (EGFvIII mock U87MG cells) after TMZ treatment. To this purpose, we performed treatment with increasing concentrations of TMZ (ranging from 0.1 to 1 mM) for different times of incubation (from 24 h to 72 h), and we evaluated cell viability using the MTT assay. As shown in Figure 1A, the treatment with TMZ for 24 h or 48 h did not significantly impair cell viability in both EGFR+ and EGFR− cells. Extending the incubation period to 72 h reduced cell survival in a concentration-dependent manner only in EGFR− cells, showing a 26%, 35%, and 86% decrease in cell viability at 100, 200 µM, and 1 mM TMZ, respectively (Figure 1A). Conversely, treatment with TMZ up to 200 µM did not significantly affect EGFR+ cell viability but increasing TMZ concentration to 1 mM significantly reduced cell survival like to that in the EGFR− cells (84% decrease) (Figure 1B). The IC50 of TMZ in EGFR+ cells was significantly increased compared to EGFR− cells (462.7 µM vs. 3062 µM), thus indicating that EGFRvIII expression is related to a reduced sensitivity to TMZ in U87 GBM cells. These data demonstrated that TMZ decreases cell survival in EGFR− cells, and the expression of EGFRvIII seems to protect the cells from the toxic effect of the drug, conferring a TMZ-resistance phenotype.

### 2.2. Sphingolipid Content Is Modified in EGFR− and EGFR+ Cells Treated with TMZ

**Figure 2:**
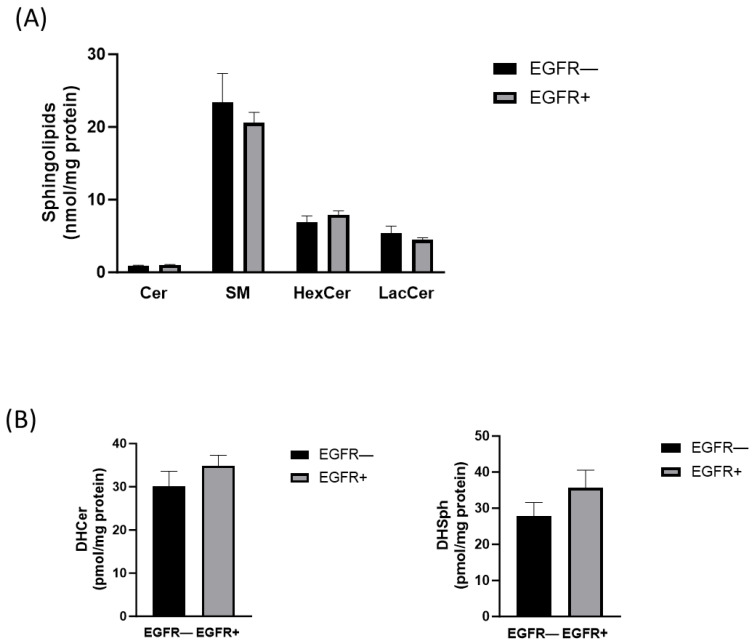
LC–MS/MS analysis of total Cer, SM, HexCer, and LacCer in EGFR− and EGFR+ cells. EGFR− (black bars) and EGFR+ cells (gray bars) were seeded, respectively, at 20,000 and 30,000 cells/cm^2^. LC–MS/MS measurements of cellular levels of (**A**) Cer, SM, HexCer, and LacCer, (**B**) DHCer and DHSph. Lipids were extracted and analyzed using LC–MS/MS. The data are averages of triplicate determinations and are expressed as pmol of lipid per mg of protein (one-way ANOVA followed by Tukey’s post hoc test).

An increasing number of studies demonstrate that SL metabolites are involved in cancer progression and drug resistance in GBM [4,13,29,30,31]. For this reason, we first evaluated whether the expression of EGFRvIII could be associated with a different SL cell profile. To this purpose, EGFR− and EGFR+ cells were submitted to lipid extraction followed by LC–MS/MS analysis. As shown in Figure 2, the total content of Cer, SM, hexosylceramide (HexCer), lactosylceramide (LacCer), dihydroceramide (DHCer), and dihydrosphingosine (DHSph) did not significantly differ between EGFR− and EGFR+ cells. Thus, we could conclude that EGFRvIII overexpression by itself did not alter the expression of the main cell SL.

**Figure 3:**
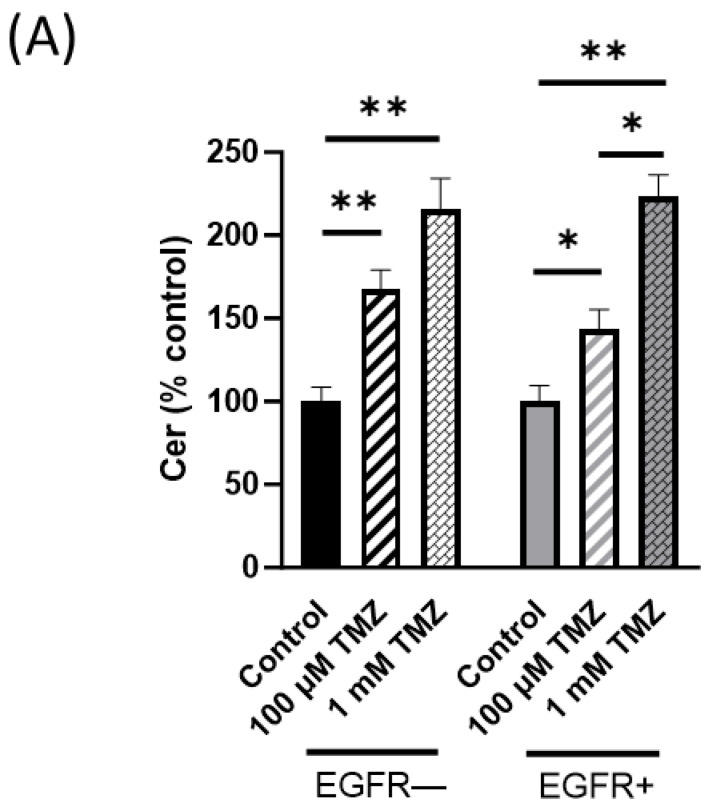

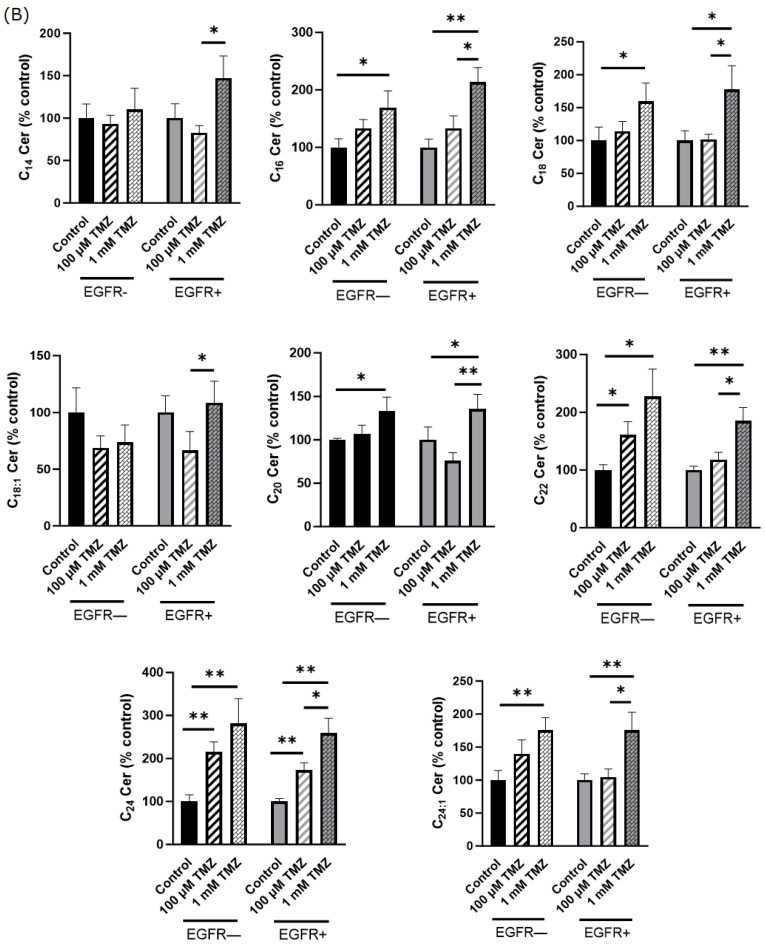

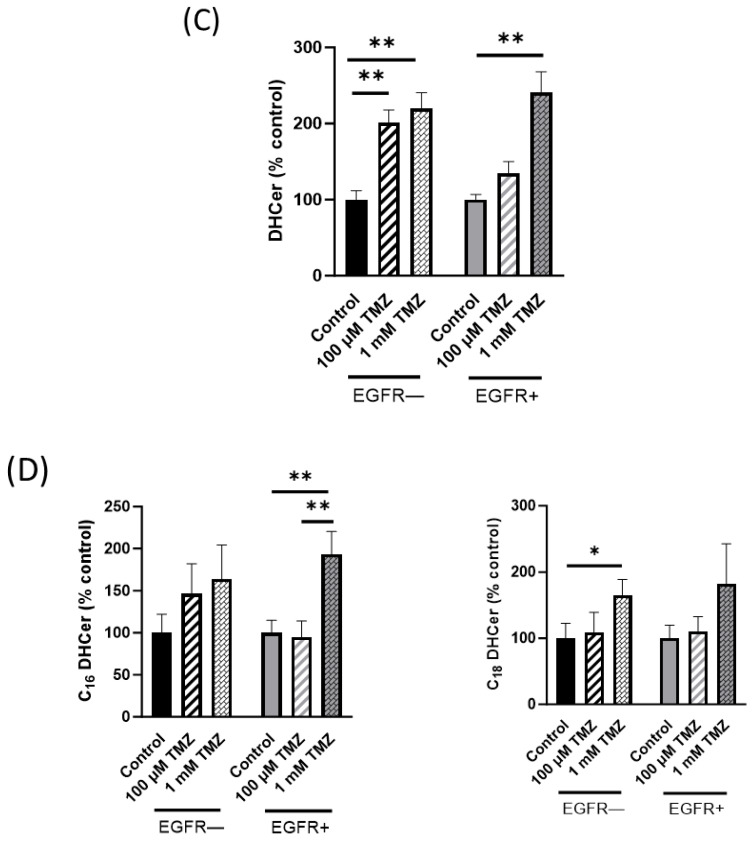

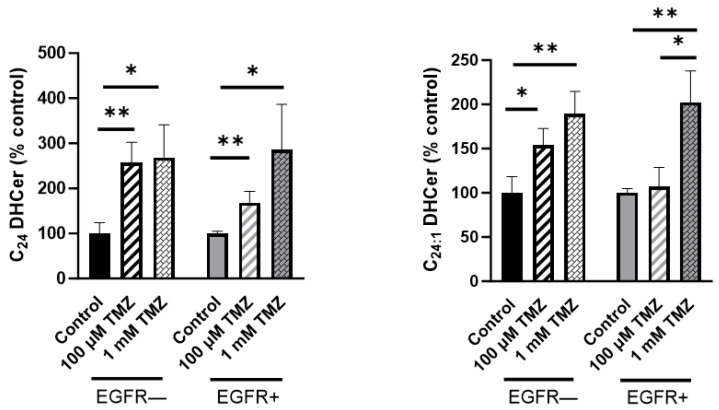
Effect of TMZ on cellular levels of Cer and DHCer in EGFR− and EGFR+ cells using LC–MS/MS analysis. EGFR− and EGFR+ cells were seeded at 20,000 and 30,000 cells/cm^2^, respectively, and treated with 100 µM or 1 mM TMZ as indicated in Materials and Methods. Lipids were extracted and (**A**) total Cer, (**B**) Cer species, (**C**) DHCer, and (**D**) DHCer species were analyzed using LC–MS/MS. The data are averages of triplicate determinations and are expressed as pmol of lipid per mg of protein (one-way ANOVA followed by Tukey’s post hoc test). * *p* < 0.05, ** *p* < 0.01.

We then examined whether TMZ treatment could modify SL metabolites differently in EGFR− and EGFR+ cells. Therefore, EGFR− and EGFR+ cells were treated for 72 h with 100 µM or 1 mM TMZ, and cellular SL composition was then analyzed using LC–MS/MS. As shown in Figure 3A, 100 µM TMZ induced a significant increase in the total Cer content in both EGFR− and EGFR+ cells (67% and 43%, respectively, compared to the respective untreated cells). The lower increase in Cer levels in EGFR+ cells suggested that resistance of EGFR+ cells to TMZ could be associated with the ability of these cells to counteract Cer increase. The administration of highly toxic doses of TMZ (1 mM) induced a further increase in Cer levels in both cell types to the same extent (116% and 126% in EGFR− and EGFR+ cells, respectively). We also used LC–MS/MS to simultaneously distinguish Cer species with different degrees of saturation and fatty acids, as well as the various acyl chain length species of the other sphingolipids. As shown in Figure 3B, treatment with 100 µM TMZ markedly increased the amount of the very-long-chain C22:0 Cer species in EGFR− cells (61%), while the same treatment did not modify the amount of all Cer acyl chain species in EGFR+ except for a slight but not significantly increase in C16:0 Cer. Treatment with 1 mM TMZ markedly increased the amount of all Cer acyl chain species in both EGFR− and EGFR+ cells, although in EGFR− cells, the C14 Cer and C18:1 Cer species were not significantly modified (Figure 3B).

**Figure 4:**
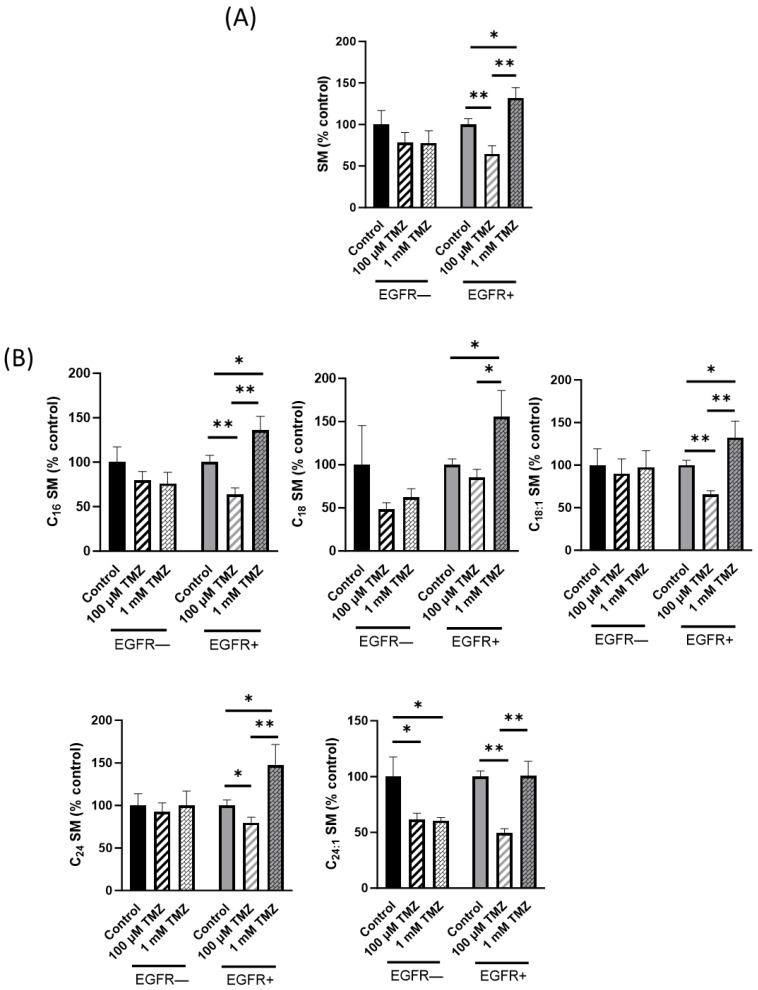
Effect of TMZ on cellular levels of SM in EGFR− and EGFR+ cells using LC–MS/MS analysis. EGFR− and EGFR+ cells were seeded, respectively, at 20,000 and 30,000 cells/cm^2^ and treated with 100 µM or 1 mM TMZ as indicated in Materials and Methods. Lipids were extracted and (**A**) total SM and (**B**) SM species were analyzed using LC–MS/MS. The data are averages of triplicate determinations and are expressed as pmol of lipid per mg of protein. * *p* < 0.05, ** *p* < 0.01 (one-way ANOVA followed by Tukey’s post hoc test).

**Figure 5:**
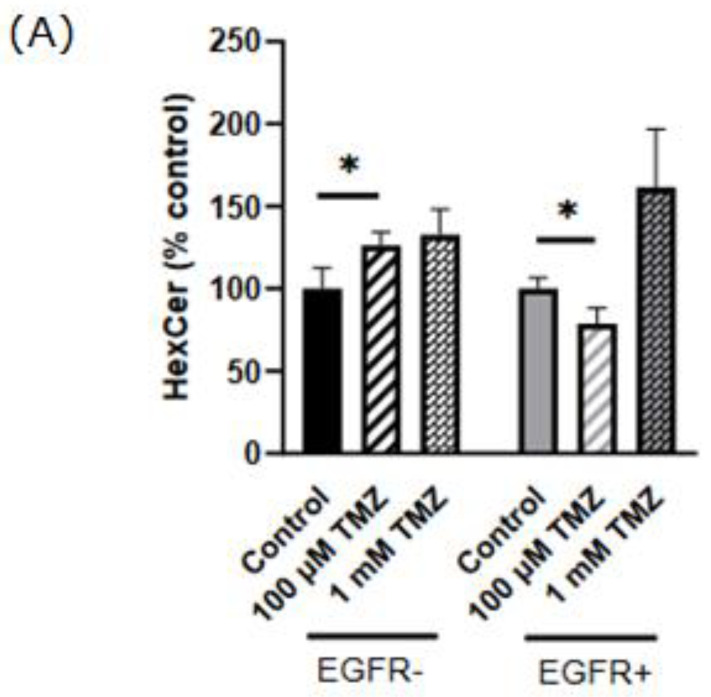

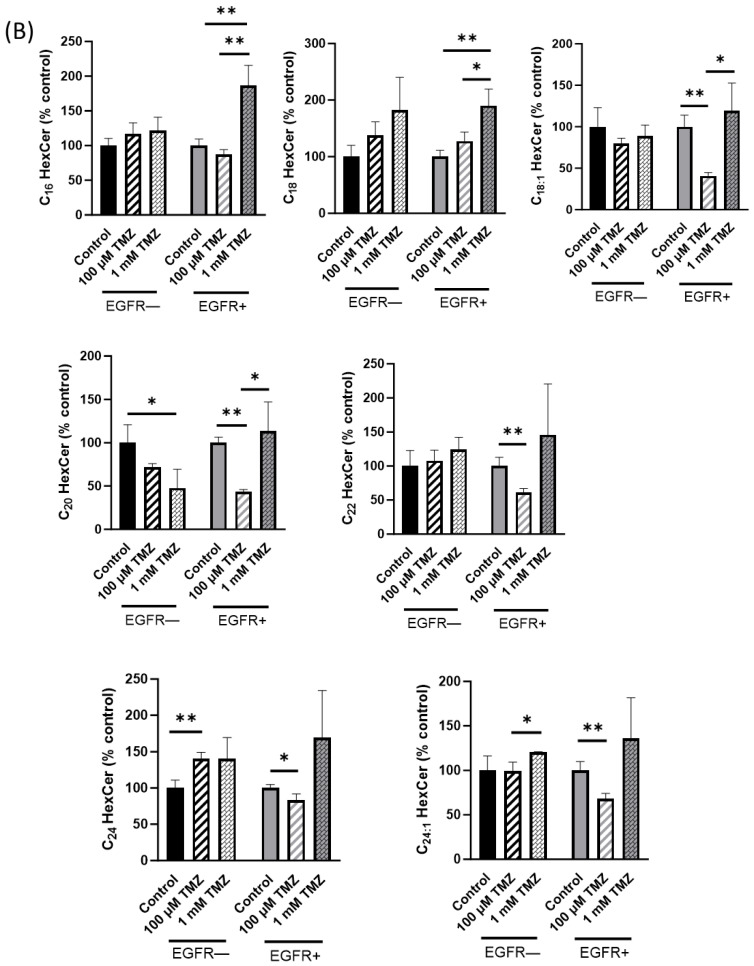

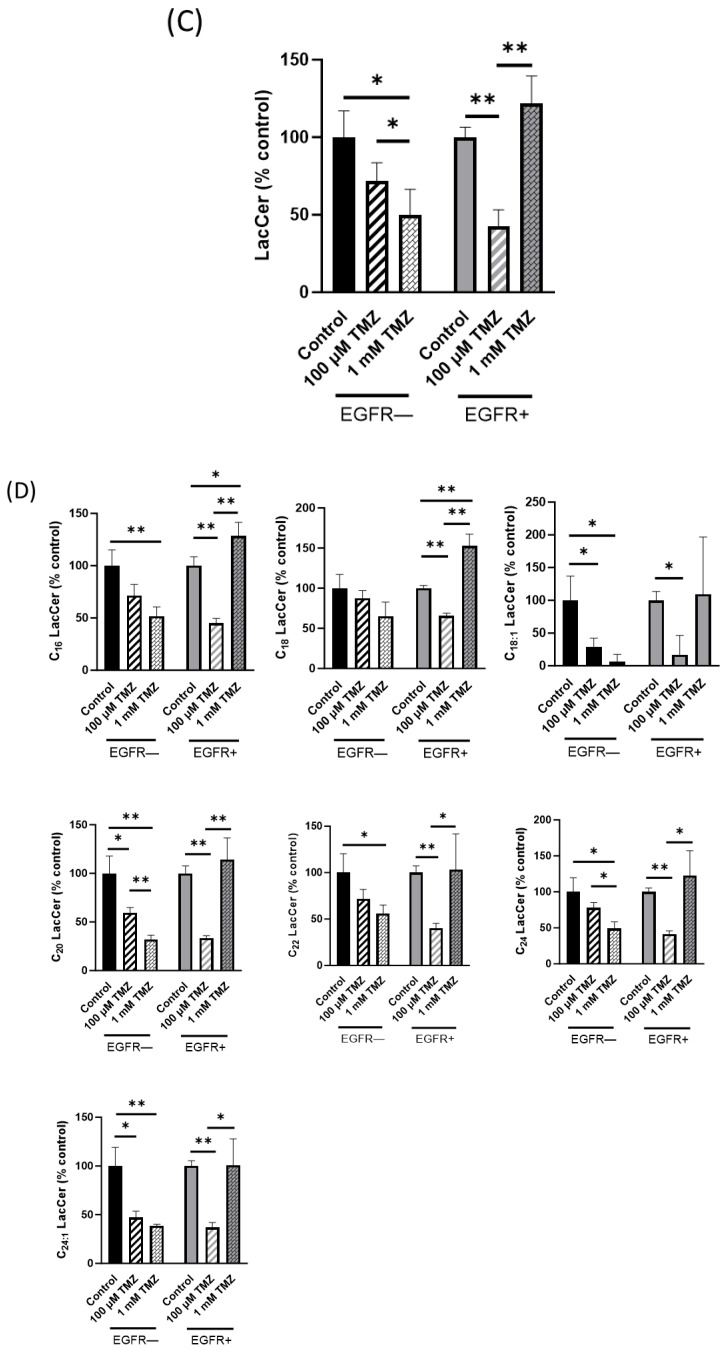
Effect of TMZ on the cellular levels of HexCer and LacCer in EGFR− and EGFR+ cells using LC–MS/MS analysis. EGFR− and EGFR+ cells were seeded, respectively, at 20,000 and 30,000 cells/cm^2^ and treated with 100 µM or 1 mM TMZ as indicated in Materials and Methods. Lipids were extracted and (**A**) total HexCer, (**B**) HexCer species, (**C**) total LacCer, and (**D**) LacCer species were analyzed using LC–MS/MS. The data are averages of triplicate determinations and are expressed as pmol of lipid per mg of protein. * *p* < 0.05, ** *p* < 0.01 (one-way ANOVA followed by Tukey’s post hoc test).

An amount of 100 µM TMZ induced a significant increase also in the total amount of DHCer in EGFR− (101%) but not in EGFR+ cells, compared to untreated cells (Figure 3C). The administration of a higher dose of TMZ (1 mM) strongly induced the increase in DHCer to the same extent in both cell types (120% and 140% in EGFR− and EGFR+ cells, respectively) (Figure 3C). Moreover, it should be noted that the treatment of EGFR− and EGFR+ cells with 100 µM TMZ did not significantly modify the amount of all DHCer acyl chain species but only C24:1 and C24 DHCer significantly increased (54% and 157%, respectively) in EGFR− cells and only C24 DHCer (68%) increased in EGFR+ cells (Figure 3D) compared to their respective control untreated cells. Treatment of EGFR− and EGFR+ cells with 1 mM TMZ markedly increased the mass of all DHCer acyl chain species, although in EGFR− cells, the C16 DHCer and, in EGFR+ cells, C18 DHCer were not significantly modified (Figure 3D). Next, we analyzed the content and the molecular species of SM, and the results obtained demonstrated that SM significantly decreased (−35.5%) in EGFR+ cells treated with 100 µM TMZ (Figure 4A). Surprisingly, 1 mM TMZ treatment significantly increased SM in the same cells (Figure 4A) compared to control cells. Treatment of EGFR+ cells with 100 µM TMZ markedly increased the levels of all SM acyl chain species, although in EGFR+ cells, C18:0 SM was not significantly modified and, in EGFR+ cells, 1 mM TMZ did not significantly modify C24:1 SM compared to control cells (Figure 4B). In EGFR− cells, the treatment with 100 µM or with 1 mM TMZ significantly decreased C24:1 SM (Figure 4B). Treatment of EGFR− cells with 100 µM TMZ markedly increased the levels of HexCer (Figure 5A) but decreased the levels of the same molecule in EGFR+ cells (Figure 5A) compared to untreated cells; 1 mM TMZ did not significantly modify HexCer in both cell types (Figure 5A).

Furthermore, treatment of EGFR− cells with 100 µM TMZ did not significantly modify the amount of all HexCer acyl chain species except for C24:0 HexCer (Figure 5B), although in EGFR+ cells, the same concentration of TMZ did not significantly modify the amount of C16:0 and C18:0 HexCer compared to untreated cells (Figure 5B) but significantly decreased C18:1, C22:0, C24:0, and C24:1 HexCer (59%, 39%, 16%, and 32%, respectively) (Figure 5B).

Finally, we evaluated the levels of LacCer, observing that the treatment of EGFR− cells with 100 µM TMZ did not significantly modify the amount of LacCer but 1 mM TMZ significantly decreased LacCer (48%); in EGFR+ cells, 100 µM TMZ treatment decreased LacCer (57%), but 1 mM TMZ did not (Figure 5C). In particular, in EGFR− cells, 1 mM TMZ significantly decreased the levels of all LacCer acyl chain species, although C18 LacCer was not significantly modified (Figure 5D); in EGFR+ cells, 100 µM TMZ treatment decreased the mass of all LacCer acyl chain species (Figure 5D).

### 2.3. Sphingolipid Metabolism Is Modified in EGFR− and EGFR+ Cells in the Presence of TMZ

**Figure 6:**
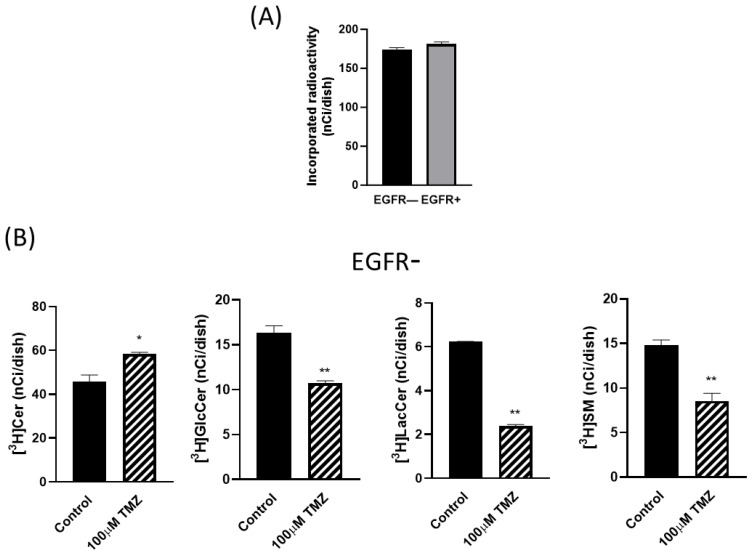

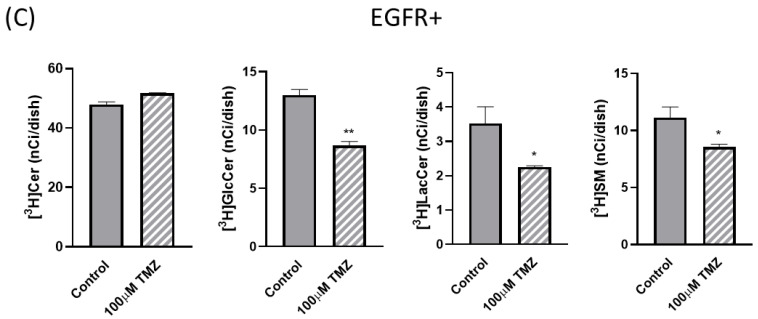
Metabolic radiolabelling of sphingolipids in EGFR− and EGFR+ cells. EGFR− (black bars) and EGFR+ cells (gray bars) were, respectively, seeded at 20,000 and 30,000 cells/cm^2^ and treated with 100 µM TMZ for 72 h. At the end of the incubation, the cells were pulsed with 20 nM [^3^H]Sph (0.4 µCi mL^−1^) for 30 min. At the end of the pulse, cells were harvested and subjected to lipid extraction and partitioning. The methanolized organic phase was analyzed using HPTLC and digital autoradiography of HPTLC as described in the Materials and Methods. Total incorporated radioactivity (**A**) and radioactivity incorporated in Cer, SM, HexCer, and LacCer in EGFR− cells (**B**) and in EGFR+ cells (**C**) are shown. ** *p* < 0.01, * *p* < 0.05 compared with control cells (*t*-test). All values are the mean ± SD of at least three independent experiments.

To evaluate the effect of TMZ on sphingolipid metabolism, EGFR− and EGFR+ cells were submitted to pulse experiments with tritiated sphingosine ([^3^H]Sph) after 72 h of treatment with 100 µM TMZ. After a short-time pulse (30 min), the incorporated radioactivity did not significantly differ between EGFR− and EGFR+ cells (Figure 6A). We then analyzed the cellular [^3^H]Cer, [^3^H]SM, [^3^H]GlcCer, and [^3^H]LacCer content. Of relevance, the amount of labelled Cer found in EGFR− cells was significantly higher (by ~27%) after the treatment with 100 µM TMZ (Figure 6B) and was paralleled by a strong decrease in the radioactivity associated with GlcCer, LacCer, and SM, respectively, by 35%, 62%, and 43% compared to the values of untreated cells (Figure 6B). On the contrary, in EGFR+ cells, the treatment with 100 µM TMZ did not significantly modify the amount of labelled Cer (Figure 6C) but induced a decrease in the radioactivity associated with GlcCer, LacCer, and SM to at a less extent than that observed in EGFR− cells by, respectively, 34%, 36%, and 24% compared to control cells (Figure 6C).

### 2.4. Effect of TMZ on Cell Survival after Inhibition of Cer Metabolism

Next, we tested whether the inhibition of Cer metabolism might be involved in TMZ resistance. To this purpose, we evaluated the effect of subtoxic doses of PPMP and D609, GlcCer or SM synthase inhibitors, respectively, on the cell viability of EGFR+ resistant cells after TMZ treatment.

**Figure 7:**
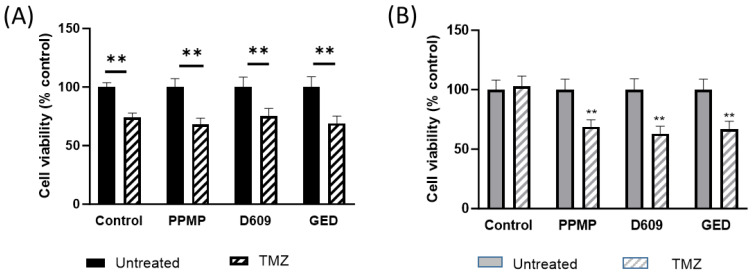
Effect of TMZ on cell survival after inhibition of Cer metabolism in EGFR− and EGFR+ cells. (**A**) EGFR− cells and (**B**) EGFR+ cells were seeded at 30,000 cells/cm^2^ and exposed to 100 µM of TMZ alone or in combination with 2.5 µM of PPMP or 2 µM of D609 or 10 µM GED. Cell viability was assessed using MTT assay after 48 h of treatment. Results are expressed as percentage of cell survival with respect to vehicle-treated cells. Data are mean ± SD of three independent experiments. ** *p* < 0.01 versus respective control (one-way ANOVA followed by Tukey’s post hoc test).

The results showed that the incubation with either PPMP or D609 at subtoxic doses (Figure S1) significantly reduced the survival rate of both EGFR− and EGFR+ cells after TMZ treatment. In EGFR− cells, PPMP or D609 together with TMZ treatment decreased cell viability by about 32% and 25%, respectively, values very similar to those obtained after incubation with TMZ alone (26%) (Figure 7A). Conversely, in EGFR+ cells, viability was suppressed only after incubation of TMZ in combination with PPMP or D609 (32% and 37% decrease, respectively), while TMZ alone did not significantly affect cell viability (Figure 7B). Moreover, in EGFR− cells, the co-treatment with PPMP or D609 and TMZ did not significantly alter Cer levels compared to TMZ alone, but in EGFR+, Cer increased after co-treatment with PPMP or D609 and TMZ compared with TMZ alone (Figure S2). Thus, the different inhibitors of Cer removal from the endoplasmic reticulum (ER) and utilization were all able to sensitize EGFR+ cells to the drug.

We next speculated that an inhibitor of CERT-mediated Cer traffic from the ER to the Golgi apparatus might augment the effect of TMZ on EGFR− and EGFR+ cells and that Cer removal from the ER might be involved in TMZ resistance. To test this hypothesis, we first analyzed the effect of gedunin, the inhibitors of CERT-mediated Cer transport from the ER to the Golgi apparatus, on TMZ toxicity in EGFR− sensitive cells and EGFR+ resistant cells. We found that the exposure of EGFR− or EGFR+ cells to a subtoxic dose of gedunin (10 µM) (Figure S1), concomitant to TMZ treatment, suppressed cell viability of about 31% (Figure 7A) and 33% (Figure 7B), respectively.

**Figure 8:**
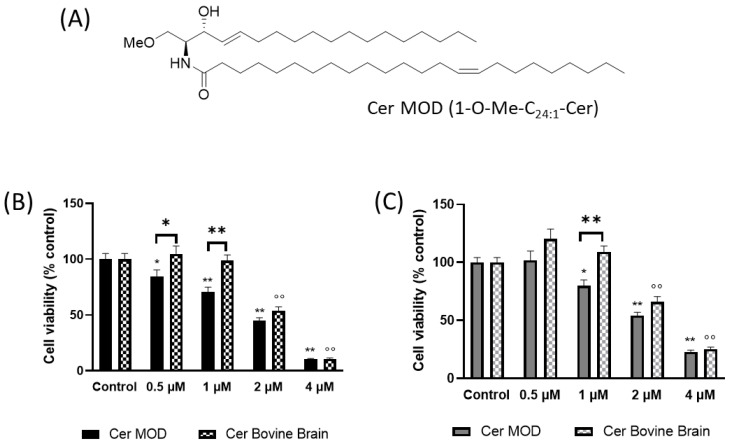
Effect of BB Cer and Cer MOD on cell survival in EGFR− and EGFR+ cells. (**A**) CerMOD structure; (**B**) EGFR− cells were seeded at 30,000 cells/cm^2^ exposed to 1–4 µM of Cer MOD or BB Cer; (**C**) EGFR+ cells were seeded at 30,000 cells/cm^2^ exposed to 1–4 µM of Cer MOD or BB Cer. Cell viability was assessed using MTT assay after 48 h of treatment. Results are expressed as percentage of cell survival with respect to vehicle-treated cells. Data are mean ± SD of three independent experiments. * *p* < 0.05, ** *p* < 0.01, ˚˚ *p* < 0.01 versus control (one-way ANOVA followed by Tukey’s post hoc test).

Since the inhibition of Cer removal from the ER to form SM and GlcCer sensitize EGFR+ cells to TMZ, we next evaluated the effect of exogenously administered bovine brain Cer (Cer BB) (composed mainly of stearic acid and nervonic acid) or a synthetic modified Cer (Cer MOD) (Figure 8A) on cell viability. We synthesized Cer MOD, methylated at the primary hydroxyl group of Cer, to prevent the ability of GlcCer and sphingomyelin synthase to metabolize Cer, thus inducing its accumulation in the ER and mimicking the situation obtained by the inhibition of Cer traffic or the block of Cer conversion to complex SL. The results showed that the incubation with either increasing doses of Cer BB or Cer MOD resulted in a significant survival reduction of EGFR− cells (Figure 8B). In particular, 0.5 µM and 1 µM Cer MOD, but not Cer BB, significantly suppressed cell viability by about 16% and 29.5%, respectively (Figure 8B). At higher concentrations (2 µM) also, Cer BB induced a decrease in cell viability (46%) as well as Cer MOD (55%) (Figure 8B). At 4 µM, both Cer MOD and Cer BB significantly reduced cell viability by 90% (Figure 8B). In EGFR+ cells, Cer MOD was able to decrease cell viability from 1 µM up to 4 µM (1 µM, 2 µM, and 4 µM decreased cell viability by 20%, 46%, and 77%, respectively), while 2 µM and 4 µM Cer BB suppressed cell viability by 34% and 75%, respectively (Figure 8C). In EGFR+ cells, 0.5 µM Cer MOD as well as Cer BB did not significantly modify cell viability (Figure 8C).

### 2.5. Effect of Temozolomide on Cytotoxicity

We next wondered whether PPMP and D609 might increase the effect of TMZ on cytotoxicity in EGFR− and EGFR+ cells. To this end, we treated both EGFR− and EGFR+ cells with 100 µM TMZ and 2.5 µM PPMP or 0.5 µg/mL D609 for 48 h and then we evaluated the LDH activity released.

**Figure 9:**
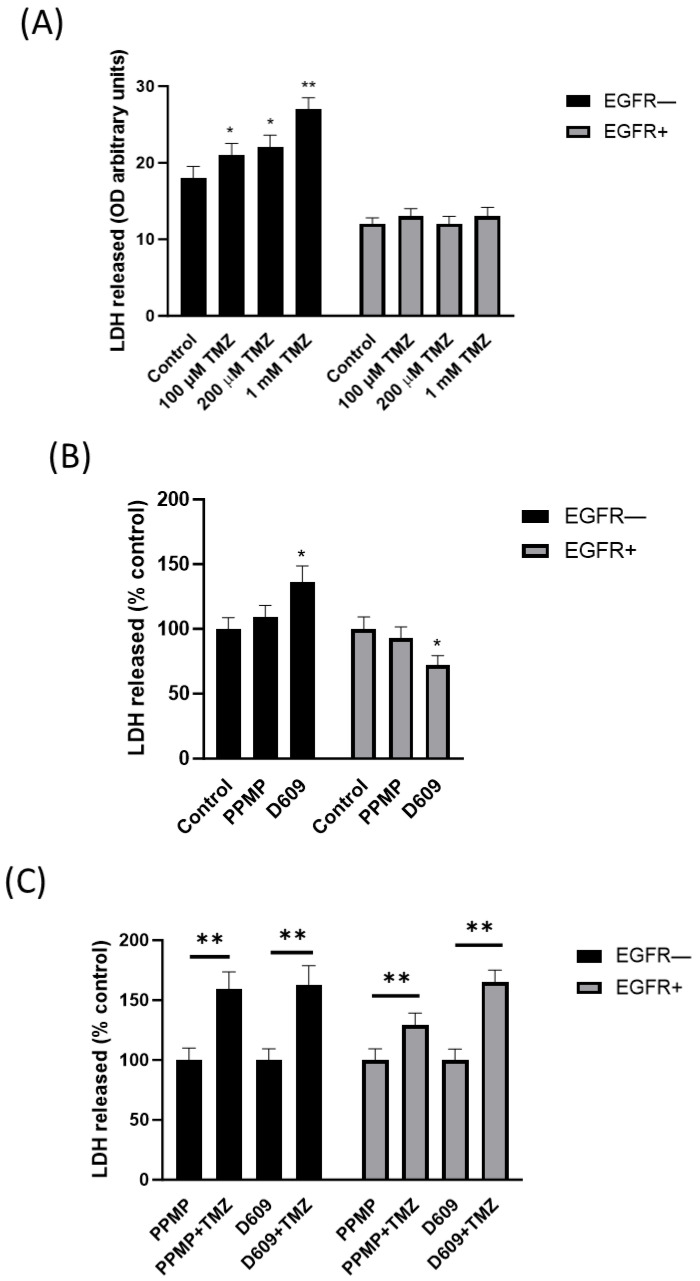
Effect of temozolomide on cytotoxicity in EGFR− and EGFR+ cells. EGFR− and EGFR+ cells were seeded at 30,000 cells/cm^2^ exposed to (**A**) the indicated doses of TMZ (100 µM–1 mM) for 48 h; (**B**) 2,5 µM of PPMP or 2 µM of D609; (**C**) 100 µM of TMZ alone or in combination with 2.5 µM of PPMP or 2 µM of D609. Cellular cytotoxicity was detected after 48 h of treatment by evaluation of the extracellular release of LDH using the LDH assay. Data are mean ± SD of three independent experiments. * *p* < 0.05, ** *p* < 0.01 (one-way ANOVA followed by Tukey’s post hoc test).

The results indicated that extracellular LDH release was increased in a dose-response manner in TMZ-treated EGFR− cells but did not significantly change at all the doses considered in EGFR+ cells (Figure 9A). Moreover, PPMP did not modify significantly the released LDH activity levels in both EGFR− and EGFR+ cells (Figure 9B), while D609 increased released LDH activity in EGFR− cells and decreased it in EGFR+ cells (Figure 9B). PPMP or D609 in co-treatment with TMZ increased extracellular LDH activity in EGFR− cells but, very importantly, in EGFR+ cells too (Figure 9C). These results suggest that the inhibition of Cer employment at the Golgi apparatus for the synthesis of complex SL-enhanced EGFR+ cells to cytotoxicity is induced by TMZ.

## 3. Discussion

Cer is an oncosuppressor lipid involved in the regulation of GBM growth [5], and it has been demonstrated that Cer levels are inversely associated with malignant progression of human glial tumors [11].

GBMs are characterized by high resistance to TMZ, the standard treatment associated with radiotherapy used for this kind of tumor [32]. Due to the poor prognosis, it is crucial to understand the mechanism of chemoresistance to develop further therapeutic strategies to find and target molecules able to increase the effect of the standard treatment. The EGFR variant known as EGFRvIII is highly specific for GBM because it is not expressed in non-tumor tissues and is present in 25–33% of all GBM patients [33]. EGFR is known to hyperactivate the phosphatidylinositol 3 kinase (PI3K)/AKT signaling pathway, which has been demonstrated to be involved in the regulation of GBM cell survival, proliferation, and motility [23]. It has been shown that EGFRvIII is constitutively active and confers a growth advantage to these tumors [23,34]. For these reasons, EGFRvIII is being studied as a possible target for therapy but not all alterations caused by it are known.

In the present study, we demonstrated that human GBM cells stably overexpressing EGFRvIII are resistant to up to 200 µM TMZ, the standard chemotherapeutic drug in GBM treatment, despite EGFR− cells. Significantly, alterations in SL metabolism seemed to be related to EGFRvIII drug resistance. Our results demonstrated that a low dose of TMZ (100 µM) significantly increases Cer levels in EGFR− cells but slightly in EGFR+ cells. Of note, 100 µM TMZ also induces a significant increase in DHCer in EGFR− cells but not in EGFR+ cells. DHCer is an intermediate molecule belonging to the de novo synthesis pathway of Cer and is directly transformed into Cer through the action of DHCer desaturase. Also, pulse metabolic experiments confirmed the acceleration in Cer synthesis pathway, identifying the accumulation of Cer in a short time in TMZ-treated EGFR− cells. These results are consistent with our previous data demonstrating that the increase in Cer levels leads to growth arrest of GBM cells [4] and when combined with TMZ treatment results in a reduction of cell viability [4].

In addition to Cer, DHCer is also involved in various biological processes such as cellular stress responses and autophagy, cell growth, pro-death or pro-survival pathways, hypoxia, and immune responses [35]. The chemical investigation of the mostly increased Cer and DHCer forms indicated that the above all long and very long chain Cers (>C22) are formed. The addition of fatty acid to the sphingoid base is catalyzed by the Cer synthases. There are six Cer synthases, each with different specificities for acyl chains, which determine the synthesis of Cer and DHCer with acyl chains of varying lengths [36]. Recent studies demonstrated that the acyl-chain length is important for Cer functions. In human colon cancer, it was demonstrated that long-chain Cers trigger apoptosis and the increase in total Cer amount was not enough to induce apoptosis without the tendency to produce very long-chain Cers [36]. The effects of the acyl length of Cers on apoptosis and cell death were mostly explained through the different interactions that can occur within the plasma membranes [37]. Thus, we demonstrated also in EGFR− cells, a disequilibrium toward the synthesis of very long acyl chain Cers occurs after TMZ treatment. The ratio between long chain (LC) Cer (C16:0) and very long chain (VLC) Cer (C22:0 and C24:0) is 0.77 and 0.56 in EGFR− and EGFR+ cells, respectively, suggesting a prevalence of pro-apoptotic vs. anti-apoptotic Cer species in EGFR− cells compared to EGFR+ cells. Furthermore, in EGFR− cells treated with 100 µM TMZ, the ratio between LC Cer and VLC Cer also indicates a prevalence of pro-apoptotic vs. anti-apoptotic Cer species (0.51 vs. 0.44). Instead, EGFR+ cells showed a minor increase in Cer levels, including very long-chain Cers. Furthermore, our results demonstrated that a mixture of long-chain Cers (bovine brain Cer) had a cytotoxic effect.

We demonstrated that EGFR+ cell viability after TMZ treatment can be decreased by compounds that increase Cer levels, PPMP, D609, and GED, which inactivate GlcCer synthase and SM synthase, respectively. Our results are in agreement with previously published data demonstrating that GlcCer synthase inhibition results in the growth arrest of GBM cells and the combination of PPMP and TMZ synergistically induces GBM cell death [4]. Also, the addition of methylated Cer had an effect in reducing cell viability. Indeed, methylated Cer that cannot be metabolized at the Golgi apparatus to synthesize SM and GlcCer, being accumulated at the ER, can decrease cell viability in EGFR+ cells at a concentration (1 µM) at which bovine brain Cer did not significantly modify cell viability.

To our knowledge, this is the first experimental evidence showing that the expression of EGFRvIII, by preventing an increase in Cer and above all very long acyl chain Cer levels, especially very long acyl chain Cers, following TMZ treatment, promotes the survival of human GBM cells. Our data demonstrated that the DHCer/Cer ratio is significantly higher in EGFR− cells compared to EGFR+ cells when the cells were treated with the subtoxic dose of TMZ (100 µM) (0.040 vs. 0.032). Therefore, it is possible to hypothesize that specific forms of ceramide synthase may have a key role in the resistance of EGFR+ cells to TMZ.

In conclusion, the results of the present study indicate that the ability of EGFRvIII to counteract Cer increase after TMZ treatment could be targeted to increase the therapy efficacy. Given the pressing need for effective GBM therapies, elucidating novel mechanisms modified in these tumors may help to identify promising targets for the development of innovative and more effective combination therapies.

## 4. Materials and Methods

### 4.1. Materials

All reagents were of analytical grade. Dulbecco’s modified Eagle’s medium (DMEM), L-glutamine, penicillin, streptomycin, amphotericin B, fatty acid-free bovine serum albumin (FAF-BSA), 3-[4,5-dimethylthiazol-2-yl]2,5-diphenyl tetrazolium bromide (MTT), bovine brain ceramide, gedunin (GED), and common chemicals were from Merck Life Science (Milan, Italy). Fetal calf serum (FCS) was obtained from Gibco-Thermo Fisher Scientific (Pero, Milano, Italy). All solvents were purchased from Merck (Darmstadt, Germany). TMZ was from Cayman Chemical (Ann Arbor, MI, USA).

DL-threo-1-phenyl-2-palmitoylamino-3-morpholino-1-propanol (PPMP) and tricyclo-decan-9-yl xanthogenate (D609) were from BIOMOL Research Laboratories Inc. (Plymouth Meeting, PA, USA).

### 4.2. Cell Cultures

U87MG human GBM cells overexpressing EGFRvIII (EGFR+ cells) (kindly provided by Prof. Pier-Luigi Lollini, University of Bologna, Bologna, Italy) or overexpressing the empty vector (EGFR− cells) were cultured in DMEM containing 10% (*v*/*v*) FCS, 2 mM L-glutamine, 100 units/mL penicillin, 100 μg/mL streptomycin, and 0.25 μg/mL amphotericin B at 37 °C in 5% CO_2_ humidified atmosphere. Stable transfectants of EGFR− and EGFR+ cells were maintained in the medium containing 1 g/L G418.

### 4.3. Cell Treatments

For cell treatments, stock solutions were prepared by dissolving the different molecules as follows: TMZ in DMSO, PPMP, and gedunin in absolute ethanol, bovine brain Cer and methylated Cer in dodecane/ethanol (2:98, *v*/*v*), and D609 was dissolved in sterile phosphate-buffered saline (PBS). Stock solutions were diluted extemporaneously in fresh medium at the desired concentrations and administered to cells for the indicated times.

### 4.4. Analysis of Cell Viability

Cell viability was determined by MTT assay. EGFR− and EGFR+ cells were seeded at 1 × 10^4^ and 2 × 10^4^ cells/cm^2^, respectively, in 96-well plates. The day after, cells were treated with different agents for the indicated periods of time. The medium was then replaced by MTT dissolved in fresh medium (0.8 mg/mL) for 4 h. The formazan crystals were then solubilized in isopropanol/formic acid (95:5 *v*/*v*) for 10 min and the absorbance (570 nm) was measured using a microplate reader (Wallack Multilabel Counter, Perkin Elmer, Boston, MA, USA).

### 4.5. [3H]Sphingosine Metabolism

Cells were plated on 35 mm dishes in the culture medium. Stock solutions of [^3^H]Sphingosine ([^3^H]Sph) in absolute ethanol were prepared and added to fresh medium. In all cases, the final concentration of ethanol never exceeded 0.1% (*v/v*). At the time of the experiment, the medium was gently removed and cells were then pulsed with 20 nM D-erythro-[3-^3^H]sphingosine ([^3^H]Sph, 0.4 µCi/mL) for 30 min, in the presence of culture medium [38]. Subsequently, cells were harvested, total lipids were extracted at 4 °C with chloroform/methanol 2:1 (*v*:*v*), and partitioned as previously reported [38]. The methanolized organic phase was analyzed by HPTLC using chloroform/methanol/water (55:20:3 *v/v/v*) as the solvent system. Digital autoradiography of HPTLC plates was performed with Beta-Imager 2000 (^T^Racer Beta-Imager, Biospace, Paris, France) and the radioactivity associated with each SL was quantified by the M3Vison software (R42-dop-m3Vision, Biospace, Paris, France). The [^3^H]-labelled SLs were recognized and identified as previously described [38].

### 4.6. Sphingolipid Extraction and Targeted LC–MS/MS Analysis

Sphingolipid extraction and targeted LC–MS/MS analysis were performed based on previously described protocols [39], with small modifications. Sphingolipids were assayed in cell suspension (100 µg protein) and collected as above described. Cells were diluted to 100 µL with water, with added 10 µL of internal standards (Cer C12:0, HexCer C12:0 and SM C12:0, 20 µM), and after the addition of 850 µL methanol/chloroform mixture (2:1 *v/v*), they were incubated for 1 h at 38 °C. Then, to enhance sphingolipid recovery, alkaline methanolysis was performed by incubation at 37° for 2 h with 75 µL of potassium hydroxide 1 M in methanol. After neutralization with 4 µL of glacial acetic acid, samples were centrifuged (25 min at 20,000× *g*) and evaporated. The residues were dissolved in 100 µL of methanol, centrifuged for 5 min at 20,000× *g*, and withdrawn in a glass vial. Finally, samples were analyzed using LC Dionex 3000 UltiMate (ThermoFisher Scientific, Waltham, MA USA) coupled to a tandem mass spectrometer AB Sciex 3200 QTRAP (AB Sciex, Framingham, MA, USA). The separation was achieved by reversed-phase chromatography using a BEH C8 1.7 μm, 100 × 2.1 mm (Waters, MA, USA) column for ceramides, dihydroceramides, glycosphingolipids, and sphingomyelins and a Cortecs C18 1.6 μm, 100 × 2.1 mm (Waters, Milford, MA, USA) column for sphingoid bases. Eluent A (0.2% formic acid 2 mM ammonium formate water-solution) and eluent B (methanol 0.2% formic acid 1 mM ammonium formate) were mixed to achieve the gradient separation. Quantitative analysis was performed interpolating each peak area of analyte/area IS with a calibration curve for each sphingolipid.

Mass spectrometry measurements was performed with the use of multiple reaction monitoring (MRM) in positive electrospray ionization for every sphingolipid here analyzed (Supplementary Tables S1 and S2).

### 4.7. Synthesis of (2S, 3R, 4E)-1-O-methyl-N-nervonoyl-sphingosine (Cer MOD, 1-O-methyl-C_24:1_-ceramide)

This compound was prepared from 3-*O*-benzoyl-*N*-nervonoyl-d-*erythro*-sphingosine, obtained after the reduction of 3-O-benzoyl-azidosphingosine [40] with H_2_S in Py/H_2_O followed by condensation with nervonic acid mediated by EDC·HCl. A mixture of 3-*O*-benzoyl-*N*-nervonoyl-d-*erythro*-sphingosine (0.020 g, 0.013 mmol) and NaH (60% mineral oil, 0.010 g, 0.26 mmol) in dry THF (1.5 mL) was stirred at room temperature for 2 h, then methyl iodide (0.010 mL, 0.12 mmol) was added dropwise. Stirring was continued at room temperature for 1 h (TLC: hexane/ethyl acetate, 1:1), then the reaction was quenched with MeOH. The mixture was diluted with water, extracted with dichloromethane, dried over sodium sulphate, and the solvents were evaporated under reduced pressure. Flash chromatography of the residue on silica gel (hexane/ethyl acetate, from 7:3 to 6:4) gave 1-*O*-methyl ceramide (R_f_ Cer MOD: 0.44 in hexane/ethyl acetate, 1:1) as a white amorphous solid (0.0055 g, 30%). ^1^H NMR (500 MHz, CDCl_3_): δ: 0.91 (t, 6H, 2CH_3_), 1.21–1.72 (m, 56H, 28 CH_2_), 2.02–2.10 (m, 6H, 2H_6_ + *CH_2_*CH = CH*CH_2_*), 2.24 (t, 2H, *C*H_2_CO), 3.36 (s, 3H, OCH_3_), 3.52 (dd, 1H, *J*_1a,1b_ = 9.7 Hz, *J*_1,2_ = 3.2 Hz, H_1a_), 3.72 (dd, 1H, *J*_1a,1b_ = 9.7 Hz, *J*_1,2_ = 3.8 Hz, H_1b_), 4.02–4.09 (m, 1H, H_2_), 4.15–4.20 (m, 1H, H_3_), 5.34–5.41 (m, 2H, HC=CH), 5.50 (ddt, 1H, *J*_4,5_ = 15.4 Hz, *J*_3,4_ = 6.0 Hz, *J_all_* = 1.5 Hz, H_4_), 5.73–5.81 (m, 1H, H_5_), 6.17 (d, 1H, *J* = 8.1 Hz, NH); ^13^C NMR (500 MHz, CDCl_3_): δ 14.1 (2CH_3_), 22.7, 25.8, 27.2, 29.2–29.8 (26 CH_2_), 32.0, 32.3, 36.8 (*C*H_2_C=O), 52.7 (C_2_), 59.3 (OCH_3_), 72.4 (C_1_), 74.3 (C_3_), 129.2 (C_4_), 129.9 (HC=CH), 133.4 (C_5_), 173.5 (C=O); ESI-MS (positive-ion mode): *m/z* = 1346.6 [2M + Na]^+^.

### 4.8. Cellular Cytotoxicity Assay by Evaluation of LDH Activity in the Extracellular Milieu

The cytotoxic effect of different molecules was assessed using a colorimetric in vitro assay that measures the extracellular release of lactate dehydrogenase (LDH). For this purpose, a commercial kit (TOX7, Sigma-Aldrich, Darmstadt, Germany) was used, which is based on the reduction of NAD by LDH. The resulting reduced form (NADH) is subsequently used in a coupled reaction, where there is the stoichiometric conversion of tetrazolium salt by a diaphorase enzyme, leading to the oxidation of NADH and the formation of a colored compound. The absorbance of the colored compound was then measured spectrophotometrically at a wavelength of 490 nm. The absorbance values are proportional to the amount of LDH released by the cells, which is directly correlated to the integrity of the cell membrane.

For each experimental condition, the amount of LDH released in the medium was evaluated as a percentage of the total cellular LDH, which was determined in parallel after the lysis of untreated control cells. The culture medium, subjected to the same experimental procedure, was used as a blank for the enzymatic reaction.

### 4.9. Statistical Analysis

Results are expressed as mean ± SD for at least three independent experiments. The statistical significance of the data was determined by the Student’s *t*-test or by the one-way analysis of variance (ANOVA) followed by the post hoc Tukey test, when applicable. Significant differences were accepted for *p* < 0.05 at least. Sigma Stat 4.0 (SysStat Software Inc., Palo Alto, CA, USA) and GraphPad PRISM 7.0a (La Jolla, CA, USA) were used for analyses and plotting.

## Data Availability

All data are available from the corresponding author on reasonable request.

## Supplementary Materials

The following supporting information can be downloaded at: https://www.mdpi.com/article/10.3390/ijms242015394/s1.
