# Supplementary material for: Ceramide Is Involved in Temozolomide Resistance in Human Glioblastoma U87MG Overexpressing EGFR
Source: Int J Mol Sci. 2023 Oct 20;24(20):15394. doi: 10.3390/ijms242015394 (PMC10607229; doi:10.3390/ijms242015394)
Supplement: Supplementary file 1 [file ijms-24-15394-s001.zip › ijms-2628222-supplementary.pdf]

Supplementary Table S1. Mass spectrometry parameters for the analysis of complex sphingolipids. In bold are reported the internal standards (IS) used for each package of lipids. Chromatographic separation for sphingolipids and glycosphingolipids is achieved on Acquity BEH C8 column [29].

Name	MRM (+)	DP (eV)	CE (eV)
**Ceramide (Cer) 12:0 (IS)**	**482.7 > 264.4**	**40**	**30**
Cer 14:0	510.7 > 264.4	40	33
Cer 16:0	538.8 > 264.4	40	36
Cer 18:1	564.8 > 264.4	40	35
Cer 18:0	566.8 > 264.4	40	36
Cer 20:0	594.8 > 264.4	40	38
Cer 22:0	622.9 > 264.4	40	39
Cer 24:1	648.9 > 264.4	40	42
Cer 24:0	650.9 > 264.4	40	33
Dihydroceramide (dhCer) 16:0	540.4 > 266.4	40	35
dhCer 18:1	566.5 > 266.4	40	35
dhCer 18:0	568.5 > 266.4	40	39
dhCer 24:1	650.5 > 266.4	40	40
dhCer 24:0	652.5 > 266.4	40	40
**Sphingomyelin (SM) 12:0 (IS)**	**647.6 > 184.3**	**40**	**29**
SM 16:0	703.5 > 184.1	60	40
SM 18:0	731.6 > 184.1	60	40
SM 18:1	729.6 > 184.1	60	40
SM 24:0	815.7 > 184.1	60	40
SM 24:1	813.7 > 184.1	60	40
**Hexosylceramide (HexCer) 12:0 (IS)**	**644.5 > 264.3**	**40**	**50**
HexCer 16:0	700.6 > 264.3	40	50
HexCer 18:0	728.6 > 264.3	40	50
HexCer 18:1	726.6 > 264.3	40	50
HexCer 20:0	756.6 > 264.3	40	50
HexCer 22:0	784.7 > 264.3	40	50
HexCer 24:0	812.7 > 264.3	40	50
HexCer 24:1	810.7 > 264.3	40	50
Lactosylceramide (LacCer) 16:0	862.6 > 264.3	60	60
LacCer 18:0	890.7 > 264.3	60	60
LacCer 18:1	888.7 > 264.3	60	60
LacCer 20:0	918.7 > 264.3	60	60
LacCer 22:0	946.7 > 264.3	60	60
LacCer 24:0	974.8 > 264.3	60	60
LacCer 24:1	972.7 > 264.3	60	60
Ganglioside GM3 (GM3) 16:0	1153.7 > 264.3	70	60
GM3 18:0	1181.7 > 264.3	70	60
GM3 18:1	1179.8 > 264.3	70	60
GM3 20:0	1209.8 > 264.3	70	60
GM3 22:0	1237.8 > 264.3	70	60
GM3 24:0	1265.8 > 264.3	70	60
GM3 24:1	1263.8 > 264.3	70	60

Supplementary Table S2. Mass spectrometry parameters for the analysis of free sphingoid bases. In bold are reported the internal standards (IS) used for each package of lipids. Chromatographic separation for sphingoid bases is achieved on Cortecs C18 column.

Name	MRM (+)	DP (eV)	CE (eV)
**Sphinganine d17:0 (IS)**	**288.4 > 252.0**	**21**	**20**
3-ketosphinganine (3KS)	300.0 > 270.4	46	23
Sphingosine (Sph)	300.3 > 282.2	21	17
Sphinosine-1-phosphate (S1P)	380.2 > 264.3	26	21
Dihydrosphingosine (dhSph)	302.2 > 284.5	31	19
Dihydrosphingosine-1- phosphate (dhS1P)	382.4 > 284.5	46	19

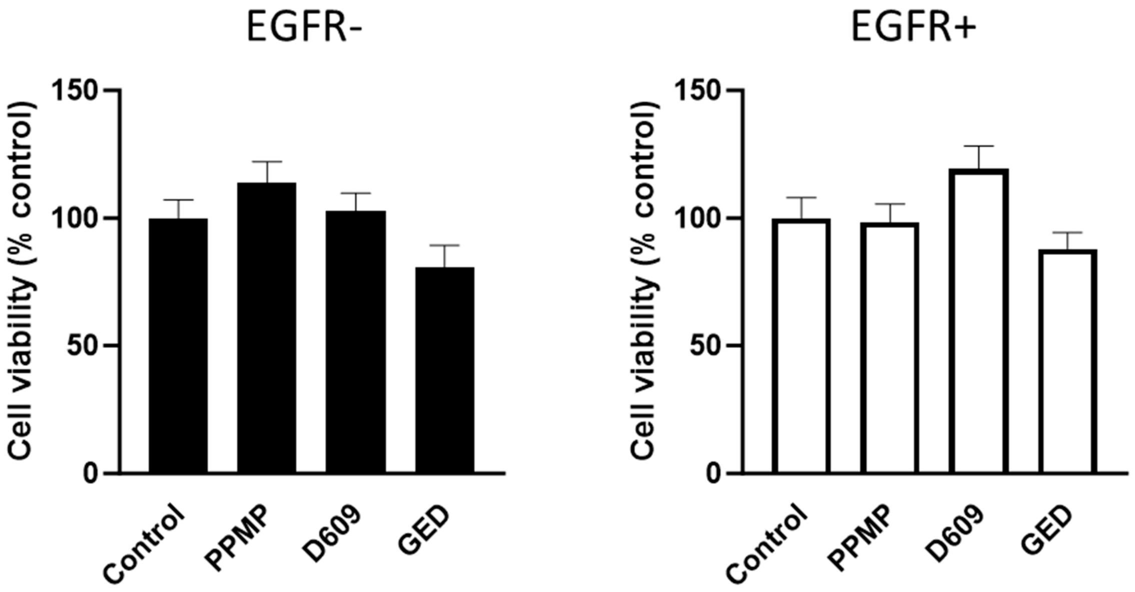

Figure S1 *Effect of PPMD and D609 on cell survival in EGFR- and EGFR+ cells*

EGFR- cells and EGFR+ cells were seeded at 30,000 cells/cm^2 and exposed to 2.5 µM of PPMP or 2µM of D609 or 10 µM of GED. Cell viability was assessed by MTT assay after 48 hours of treatment. Results are expressed as the percentage of cell survival relative to vehicle-treated cells. Data represent the mean ±SD of three independent experiments. (one-way ANOVA followed by Tukey's post hoc test).

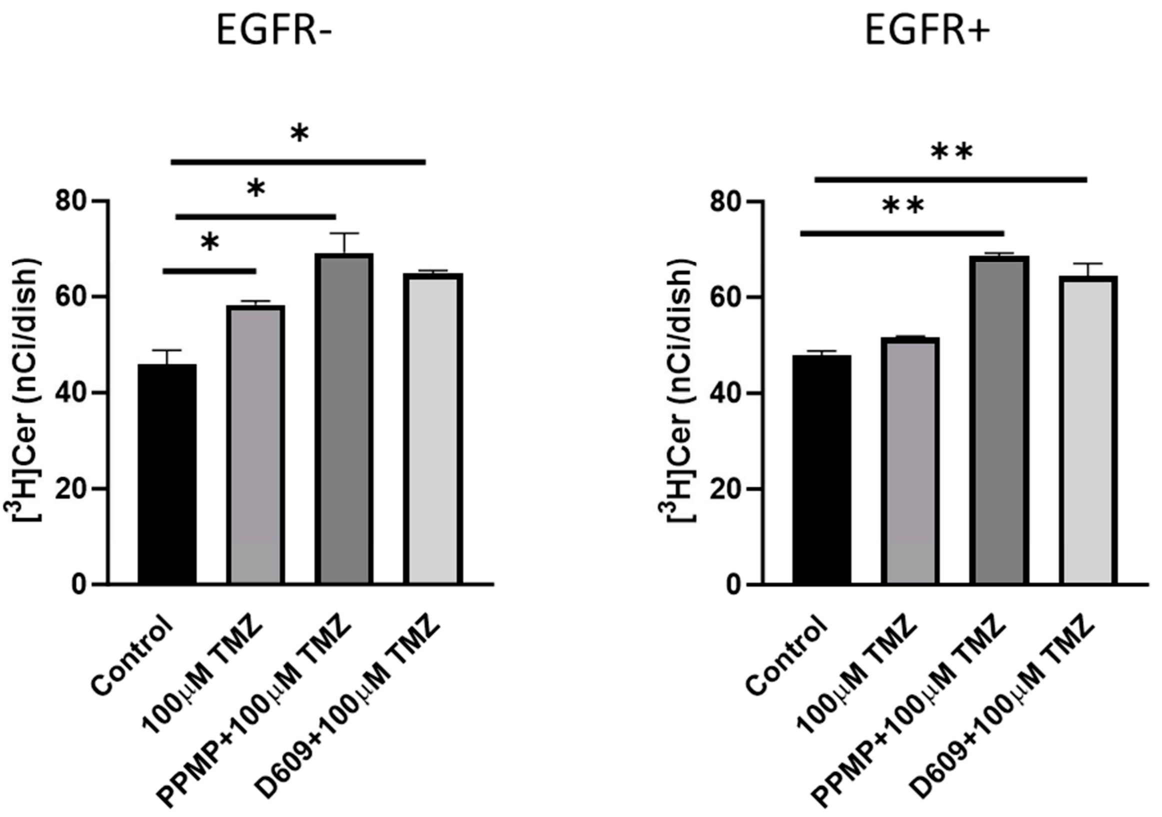

Figure S2 *Effect of PPMD and D609 on Cer levels in EGFR- and EGFR+ cells*

EGFR- and EGFR+ cells were respectively seeded at 20,000 and 30,000 cells/cm^2 and treated with 100 µM TMZ for 72 hours, either alone or in combination with 2.5 µM of PPMP or 2µM of D609. At the end of the incubation, the cells were pulsed with 20 nM [^{3}H]Sph (0.4 µCi mL^{-1}) for 30 minutes. At the end of the pulse, cells were harvested and subjected to lipid extraction and partitioning. The methanolized organic phase was analyzed by HPTLC and digital autoradiography of HPTLC as described in the Materials and methods. Radioactivity incorporated in Cer, in EGFR- cells and in EGFR+ cells is shown. *P<0.05, ** p<0.01 versus respective control (one-way ANOVA followed by Tukey's post hoc test). All values are the mean ± SD of at least three independent experiments.
